# Medical Commentaries on Puerperal Fever, Vermination, and Water in the Head

**Published:** 1836-07-01

**Authors:** 


### MEDICAL COMMENTARIES ON PUERPERAL FEVER, VERMINATION, AND WATER IN THE HEAD. By *John Alexander*, M.D. Octavo, pp. 70. Longman and Co. 1836.

### I. PUERPERAL FEVER.

Dr. ALEXANDER is physician to the Manchester General Dispensary, where he has ample field for observation and experiment. The substance of these papers has already appeared in the periodicals of the day—a circumstance that will very much limit the circulation of the present pamphlet.

We will not follow our author through the history of puerperal fever, from Hippocrates to the present hour, as we consider such procedure a waste of time. Our author conceives that much of the discrepancy of opinion respecting the nature and seat of the disease, has arisen from the confounding of different affections under the same head. Accuracy of discrimination is, therefore, of the greatest importance.

"From two to seven days after an accouchement apparently favourable, the woman is sensible of a chill, sometimes amounting to rigor or shivering, over the surface of the body ; and complains of a degree of weight, occasionally of pain, over the forehead. Her mind is anxious and irritable ; her countenance unaccountably saddened ; her utterance, pulse, and respiration, are quickened. She perceives an oppression at the præcordia, together with an uneasy general sensation throughout the abdomen, which is more or less tumid, but not at first particularly painful. There is often observed a singular apathy respecting the child. Upon the state of the various secretory functions being inquired into, we find the cuticular, lacteal, renal, and uterine secretions, all more or less suppressed. Accompanying these symptoms, the collapse of the whole animal system is as remarkable in most cases, as it is sudden. Should these characteristics of Puerperal Fever increase in degree, and assume the malignant or adynamic type, the poor sufferer, in a very short time, sinks into the arms of death. If, however, the character of the attack be otherwise, and the woman's strength prove equal to supporting her through the above state, she is generally attacked by what writers have termed *the second stage* of this complaint. Tinnitus aurium ; vomiting of bilious thin coffee-coloured matter, or occasionally of a porraceous saburra, present themselves. The tongue is observed in two very different states, with a pale or very red aspect. There is a severe and constant pain in some one of the abdominal regions ; or a pain, of a less acute nature, extensively diffused throughout the distended abdomen, having, in many cases, the peritoneum apparently for its seat. The pulse, in addition to rapidity and smallness, assumes a sharper character. Dr. Blundell says he has seldom found it under 115, unless the disease has been yielding. He has counted it as high as 165 or 170. My experience would rate its *general* frequency at about 120. The secretions continue suppressed, the alvine excepted. Singular to remark, the patient, at this crisis, previously depressed, often becomes sanguine of recovery. Provided the complaint run its too usual course, the ill-fated woman rapidly passes into the *last stage*, recognized by dull eyes with dilated pupil, sharpened nose and hectic flush (as in typhus), more or less complete and sudden alleviation of the abdominal pain, feeble fluttering pulse, involuntary excretions, hiccough, and cold extremities, the usual heralds of dissolution." 10.

The appearances on dissection vary, according to the duration of the disease. If the patient be cut off early, the post-mortem phenomena will be very indefinite. If the fever runs on for some time before it destroys life, many lesions take place, which, however, can hardly be looked upon as other than the effects, not the causes of the fever. The following is the pathological theory, or the theoretical pathology, of our present author.

"What is Puerperal Fever? I conceive it to be *a depressing affection of the abdominal nerves (as well as, in less degree, of the nervous system generally), strictly sui generis;* of which, from the peculiar nature of their functions, we are unable to take any physical cognizance beyond such as relates to the observance of their morbid influences on the human frame. I apprehend it to be perfectly distinct from an inflammatory affection (its almost universally reputed character), although frequently terminating in, or leading to, one. And for this belief my reasons are as follows:—Physiology has long ago proved that, although the nerves are not the organs of secretion, they superintend that process, as they do the equally important one of generating animal heat. Now, in this peculiar affection of the female economy, what are the symptoms presenting themselves, in what authors term the first stage? They are general chilliness of the whole system, suppression of the several secretions, and diffused uneasiness throughout the abdomen. When to these are superadded the symptoms of pain in the frontal region, uneasiness of the mind, and sudden muscular depression (almost invariable concomitants), we have abundant and, I conceive, conclusive evidence of some morbific influence exerting itself upon the (affirmed) primary seat of the malady—the nervous system. The resulting affection is extremely severe in these cases, because the part more especially attacked, the abdominal portion of that nervous system, is most important in itself; exerts extensive sympathies, and the puerperal condition is peculiarly favourable to the origin and powerful influence of morbific action." 14.

We think these views will, with very little straining, apply to any fever —or, indeed, to any disease. No doubt the nervous system takes the initiative in almost every morbid process in the living machine; but the vascular system is very quickly drawn into the circle of disorder, and must always be taken into account. Dr. Alexander, indeed, admits that "puerperal fever, if it do not rapidly prove fatal, generally merges into an inflammatory affection, commonly termed its second stage."

"The inflammation may attack the peritoneum, the ovaria, the uterus or the intestines, all at once, successively, or be confined to one organ. The particular part attacked has appeared to me determined by circumstances. Should the intestines have been unduly loaded, they suffer; if the preceding labour has been abnormal, the uterus; provided cold has been the excitant, very often the peritoneum; in short, there is great variation in the seat of the inflammation, and hence, it is more than probable, has arisen the greatest cause, as it is the most natural one, of discrepancy amongst writers upon the pathology of Puerperal Fever." 16.

When called to a patient labouring under the disease in question, Dr. A. examines the abdomen, and if the uneasiness be general, and not increased on pressure, the whole of the bowels should be covered with a large bran poultice, thinly laid, but constantly applied as hot as can be borne. To blisters he is decidedly opposed. The bowels he relieves by an enema, containing colocynth and soda. If called early Dr. A. has bled—though not largely—and with apparent benefit—even when there only seemed an inflammatory diathesis—but still more so when there existed actual local inflammation. Our author, following the sentiments of Burns, contends that puerperal fever is not to be treated like common peritonitis. Calomel and Dover's powder he always found to "act beneficially—often admirably."

"*These medicines must, however, not be exhibited in the usual dose, but given largely; indeed their exact quantity should be regulated* ONLY *by their effects, the practitioner's discretion, and the intensity or mildness of the attack.*" 23.

His favourite and usual aperient, in this disease, has been a combination of oil of turpentine and castor-oil, in equal quantities. When symptoms of sinking appear, spiced wines, burnt brandy, æther, and other diffusible stimuli were given.

We have thus given the sum and substance of Dr. Alexander's brochure, as far as puerperal fever is concerned. With the exception of leeches, which he does not mention, the treatment differs not materially, we think, from that which is generally adopted by most practitioners.

### II. VERMINATION.

Dr. A.'s attention was first drawn to this subject, about six years ago, in consequence of seeing a child, five years of age, affected in a most severe degree by convulsions, which resisted every mode of treatment till a lumbricus teres was expelled, when the child sank into a profound sleep, and no more convulsions occurred. No worms were ever passed before or after, though anthelmintics were administered. The cause of intestinal worms is wrapt in impenetrable darkness. They have been attributed to a weakened state of the alimentary canal—to ova carried into the stomach—to a peculiar state of the intestinal secretions (which means nothing)—to gastro-enterite (Broussais)—to equivocal generation.

In respect to treatment, the ascarides are often destroyed and dislodged by aloetic injections, followed by purgatives.

"The symptoms attendant upon the presence of the *cucurbitina,* or flat short worm, are much more numerous, and of a more general character. The nasal and anal pruritus are less perceived, but nevertheless present; the temper is irritable, and the child subject to grinding of the teeth, starting, and low moaning during its sleep. The countenance is of a pasty look; the skin yellowish; the eye dull, and frequently surrounded with a darkish areola; the lips and nostrils are thick, swollen, and apparently œdematous; the breath in a morning is fetid; the abdomen tumid, more or less hard, and, in the umbilical region, frequently painful. A sudden and severe pain at the epigastrium is a common symptom of Vermination; as also are griping pains, suddenly arising and as suddenly subsiding. The child's urine is observed in two states, very plentiful and pale; or, what is much more usual, scanty, thick, and of a milky appearance. The fæcal dejections are extremely irregular; at one time scybalous, at another diarrhœal, but always accompanied by more or less slimy and offensive mucus. This form of disorder is so common in the nursery, that I need not extend its description, but shall proceed to the *treatment* required for its removal.

First, then, as to the child's *diet.*—Fresh vegetables, pastry, sweets, and salted meats should be altogether interdicted. Stale wheaten bread, arrow-root or sago, with milk, should form the evening and morning meal; fresh animal food, such as mutton-chop, beef-steak, or boiled fowl, *in small quantity,* the meridian or noon repast. To these, by way of change, may be added the various animal broths, and a little good rice. It may be—nay frequently is—objected to this dietary, that the hitherto indulged child will not be contented with it. My answer is (and the mothers's answer should be)—let it fast until it will! The period of abstinence is seldom a protracted, always a beneficial one. In dispensary practice, the cure of verminative disorders is constantly impeded by the parent being utterly unable to procure appropriate food for the child; and, owing to this too common occurrence, we are rendered painfully sensible of the great importance of a regulated diet in Vermination.

Next, as to *medicine*.—It has been my custom to commence the treatment by administering scammony, jalap, and calomel, in small doses, suited of course to the age, every alternate night at bed-time; succeeded, the mornings following, by a moderate quantity of castor-oil. There are two reasons which I conceive render small doses of aperients in this complaint preferable. They have a better opportunity of exerting their alterative effect, by being leisurely passed through the alimentary tube; and, secondly, in the great majority of verminative cases which I have witnessed, the little sufferer has been more or less troubled with partial prolapsus of the gut after defecation—and it is scarcely necessary to add, that powerful purgatives render this local debility a great source of distress. At the same time I fully concur with my esteemed friend and Dispensary colleague, Mr. Stott, in thinking that the *first* purgative should be in full dose, as a free evacuation of the large intestines gives marked relief to the accumulative distention and bearing down, that attend upon these cases. Gamboge, from being tasteless, would constitute an aperient peculiarly adapted for children, but it is a drug which, sometimes, not merely occasions considerable sickness, but signally fails in answering the object we have in view; hence I cannot recommend, as many have done, its adoption. After the first passages have been *daily* unloaded for a week, the spirit of turpentine mixed with castor-oil may next be given with the most salutary and marked effect, not unfrequently removing hundreds of these enemies of childhood at every dejection." 33.

### III. Acute Hydrocephalus.

This is defined by our author thus:—" a specific affection of the brain, generally leading to serous or lymphatic effusion." The malady runs through three stages—the congestive, inflammatory, and effusive—to which Golis has added a paralytic stage. The reasoning and the remedies of our author present nothing novel. They are a kind of sensible and judicious medium between extremes.

